# Establishment of medical education upon internalization of virtue ethics: bridging the gap between theory and practice

**Published:** 2017-04-05

**Authors:** Mansoureh Madani, Bagher Larijani, Ensieh Madani, Nazafarin Ghasemzadeh

**Affiliations:** 1 PhD Candidate in Medical Ethics, Medical Ethics and History of Medicine Research Center, Tehran University of Medical Sciences, Tehran, Iran.; 2 Professor, Medical Ethics and History of Medicine Research Center, Endocrinology and Metabolism Clinical Sciences Institute, Tehran University of Medical Sciences, Tehran, Iran.; 3 Lecturer, Department of Islamic Knowledge and Humanities, Amirkabir University of Technology, Tehran, Iran.

**Keywords:** Medical education, Virtue ethics, Moral emotions

## Abstract

During medical training, students obtain enough skills and knowledge. However, medical ethics accomplishes its goals when, together with training medical courses, it guides students behavior towards morality so that ethics-oriented medical practice is internalized. Medical ethics is a branch of applied ethics which tries to introduce ethics into physicians’ practice and ethical decisions; thus, it necessitates the behavior to be ethical. Therefore, when students are being trained, they need to be supplied with those guidelines which turn ethical instructions into practice to the extent possible. The current text discusses the narrowing of the gap between ethical theory and practice, especially in the field of medical education.

The current study was composed using analytical review procedures. Thus, classical ethics philosophy, psychology books, and related articles were used to select the relevant pieces of information about internalizing behavior and medical education. The aim of the present study was to propose a theory by analyzing the related articles and books.

The attempt to fill the gap between medical theory and practice using external factors such as law has been faced with a great deal of limitations. Accordingly, the present article tries to investigate how and why medical training must take internalizing ethical instructions into consideration, and indicate the importance of influential internal factors.

Virtue-centered education, education of moral emotions, changing and strengthening of attitudes through education, and the wise use of administrative regulations can be an effective way of teaching ethical practice in medicine.

## Introduction

The goal of medical education programs is to train physicians and provide them with the skills and knowledge required to perform their duty in a legal and ethical framework, because a proficient physician who is knowledgeable enough will not achieve his goal, which is improving patients’ health, if he practices medicine in an unethical way. With regard to this issue, teaching medical ethics and the related laws has been emphasized in the form of important curricula and nurturing ethical physicians (1). Regardless of the theoretical basis it is founded upon, ethics is closely related to practice. Ethical systems try to present a model to modify and correct individuals’ behavior with respect to every taste and style (2). 

Applied ethics is a branch of ethics that involves the application of ethics in the private and public lives of people and tries to solve common tangible problems in a variety of communities and professions. Applied ethics does not aim at developing the understanding of ethical concepts, but at making suggestions that have beneficial practical results and assists in ethical decision-making (3). Medical ethics is considered to be one of the branches of applied ethics. Its orientation is towards tangible medical issues and it intends to explain the principles and basic concepts of this science and introduces ethics into the field of practice and important ethical decisions (4). In this vein, medical ethics focuses on putting ethics into practice in real-life situations. It is very clear that this branch will succeed if it puts the findings of theoretical research into practice and accomplishes its practical aims. This objective raises the issue of the relationship between knowledge and practice.

People’s voluntary behavior (that is, the topic of ethics) is shaped on the basis of their willingness and determination. It is evident that voluntary behavior is preceded by a sort of science and cognition, but not every science and theory necessarily leads to practice. We are always dealing with behavior patterns that are in contrast with the beliefs and ideas of their owners (2). Many people who have dangerous behavior, like people who smoke or diabetics who do not follow their diets, believe that their behavior is hazardous and inappropriate. Furthermore, regarding ethical behavior, it can be stated that many physicians who do not respect patient’s rights confess that their behavior is wrong or at least are aware of their false behavior from the viewpoint of medical ethics. 

In order to clarify this issue, it is essential to study the factors that lead to or prevent the transformation of knowledge or beliefs into behavior which has previously been studied in various areas. These studies have mainly considered converting an idea into practice as a process and the stages between these two, and have studied the effect of each. These stages are not identical in various fields, but they have many similarities and the intermediation of desire is accepted in almost all of these models, although it has different names such as attitude, intention, and motivation. Regardless of any of these theories, it can generally be mentioned that whatever obliges an individual to act can be extrinsic or intrinsic. The extrinsic factor can be law or custom (5). Law is one of the tools that are employed to control the individual, and professional and organizational behavior. Although it is powerful, it is inefficient to some extent. For instance, it is costly, and unable to enter some individual and social domains, including private relationships, and control some behavior. Like law, custom also relies on external enforcement such as people’s humiliating and blaming looks. Therefore, focusing on internal factors would lead to more favorable practical outcomes.

Internalization of behavior starts from childhood and characters are established gradually. When an individual enters the medical training field, he/she starts learning new knowledge and skills and uses this knowledge in his/her behavior. Some of these trainings are imposed on the individual by administrative and regulatory requirements, and in the event of a malfunction or failure of the regulatory system, the individual refuses to act accordingly. However, other trainings turn into a personal value system. In this vein, it is considered that the efforts of the medical education system regarding the presentation of high quality education which results in internalization of most of the teachings will end in greater practical success.

On the other hand, teaching ethics cannot be separated from medical training and medical students must be trained in a way that they acquire the knowledge of both ethics and medicine. Furthermore, medical training curricula are required to provide the necessary skills and to give an assurance for dealing with challenging problems of medical groups (1). In this regard, universities should pay greater attention to the roles of administrative actions, educational settings, and teachers as role models for students. Moreover, they should center their education on training more professional-oriented physicians (6), who have greater intrinsic motivation and do not require external control.

Due to the fact that it is a new subject area, few studies have been conducted to investigate the issue. The aim of the current study was to illustrate that in order to narrow the gap between ethical theory and practice, especially in the field of medicine, it is crucial to consider behaviors determining intrinsic factors and find ways to intervene in these behaviors, and consequently, change them through medical education.

## Method

The current article was composed using analytical review procedures. First, the terms internalization, medical education, ethical education, and virtue ethics were searched using valid scientific websites such as INLM, PubMed, and Google Scholar. A combination of keywords was used in the search strategy. Moreover, classical ethics philosophy and psychology books were used to select the relevant pieces of information. Other related books were also found and utilized using these keywords and related articles were identified on the basis of the booklist. After the meticulous studying of the books and articles, the issue of virtue ethics internalization and medical education was discussed. 

## Results

The main effective factors on ethical internalization and some of the important interventional manners by which the gap between ethical theory and practice can be narrowed are summarized in table 1. 

**Table 1:** Effective factors on ethical internalization and interventional manner

**Effective Factors on Ethical ** **Internalization**	**Intervention in Medical Education**
Role of Emotions in medical decision making	Nurture of students' moral sentiments Consideration of emotion's cognitive component and its promotion through medical education Creation of sublimation emotions in medical students Teaching empathy to medical students
Moral and virtuous character	Nurture of characteristics such as strong will, self-confidence, self-motivation, and a sense of control over oneself in medical school Instilling of a moral and virtuous character in medical students and creation of moral agent or teaching of virtue through professionalism education Careful consideration of hidden curriculum and teachers as role models in medical education
Attitude and change of attitude	Consideration of the cognitive, affective, and behavioral dimensions of attitude in medical education Creating attitude and changing attitude through medical education Creating and strengthening positive attitudes Use of faith for the desire to act
Appropriate application of law and administrative Solutions	Codification of appropriate disciplinary regulations (neither strict and nor permissive) with stakeholders involvement and based on their moral convictions and physical and spiritual needs Ethical justification of medical regulations or ethical guidelines and creation of a cultural infrastructure in the medical education system Administrative solutions for subordination of regulations (appropriate authority) Education of self-control and self-regulation for medical students

**Figure 1:**
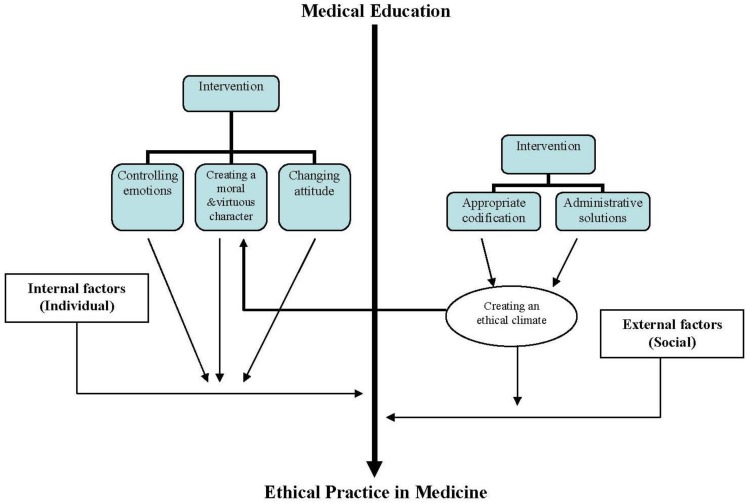
Internalization of ethical instructions in medical education

The main findings of this study will be discussed in detail in subsequent sections. In this regard, medical schools must plan for internalization of ethical instructions in medical education based on appropriate interventions on internal and external factors (Figure 1).

**Particular importance of internal requirements in modern medicine**

The definition of “ethics” in modern medicine differs entirely from its classical meaning. Traditional medical ethics was deeply rooted in the ideas of great philosophers such as Socrates and Plato. For them, ethics was the art of living and a method of self-care which they were trying to learn in addition to medicine (7). After the Renaissance, religious teachings were criticized and rationalist orientations increased, especially in the west, and accordingly, ethical attitudes were influenced. Even in the late 19^th^ and 20^th^ century, originality was completely directed toward the human biological identity and physical pleasures with the rise of theories like the theories of Freud and Darwin (8).

In recent years, the scholars of bioethics have greatly criticized the common structure of modern medicine and medical ethics. George Engel, who is one of the most prominent critics, believes that the biomedical approach has mostly emphasized the cellular and molecular level of the individual’s characteristics; however, we deal more with patients’ personal dimension rather than their cellular and molecular identity (9). In modern medicine, the biomechanical model is the basis for research, education, and treatment. As a result, physicians unconsciously conceive patients as machines and their illnesses as mechanical defects. In this way of conception, the physician does not have the ability to deal with patients’ underlying life issues, and thus, cannot have an appropriate relationship with them. Therefore, in order to introduce ethics into medical contexts, it is essential that this attitude toward human beings is revised (10).

Von Foerster, another critic of modern medical ethics, states that achieving ethics in life cannot be accomplished through ethical orders (11). He believes that, in the context of life, ethics is related to the time when an individual decides to do or not to do, whereas ethical imperative tells us what to do or what not to do (11).

Another critic is Ten Have who notes that, after 1960s, medical ethics, which previously was more like internal ethics, has distanced from its traditional deontologist form and has appeared as modern bioethics in the shape of some applied principles and rules (12). In addition to criticizing, he expressed sorrow for this historical trend and the trade-like nature of modern medicine and points that the transformation trend of internal professional ethics into external applied ethics is a stranger to its clinical reality. In his opinion, an appropriate interaction must be sought between traditional medical ethics and modern bioethics (12).

In Iran, medicine is also moving towards this modern structure, while in the past, before and after Islam, medicine was closely connected to wisdom (*Hekmat*) and theology. Especially after Islam, Iranian physicians paid specific attention to medical ethics due to the great Islamic emphasis on ethical issues because the promotion of human ethics is the ultimate goal of the prophets’ mission (*Be’sat*). In Islam, medical ethics is closely related to *Fiqh* and *Sharia* according to which the ethical nature of an action is not only related to the characteristics of that action itself, but also to the agent’s qualities. Therefore, in practical terms it employs consistent principles in clinical decision making, although it underscores piety and moral virtues and is a kind of “virtue-based ethics” (13).

**Importance of intrinsic motivation and requirement in medicine**

In the field of medicine, there are numerous situations in which a person decides without thinking and only on the basis of his own intuitions and internal requirements. One such situation is when the physician decides immediately and impromptu or when he/she reacts unconsciously which is very important in the field of medicine since a physician is repeatedly faced with such situations that are both sensitive and vital and there is the need for urgent decision making, too. In these situations, the individual unconsciously uses her/his previously internalized patterns that are externally more difficult to monitor. In this stage, ethical reasoning intervenes less (14-16), and the individual’s characteristics, ethical sensitivity, and emotions are more determining; thus, the best ethical results are gained when ethical instructions have been internalized within the individual. Internalizing ethical instructions is important in the case of delayed decision makings, especially when the individual experiences emotions and excitements which, in turn, decrease the power of logic and wisdom (14). 

Complex ethical situations are other cases in which the physician makes a decision based on his intuition and internal motives. Some ethical problems are so complicated that their solutions inevitably require a moral agent who has an ethical character. Therefore, the individual can only make the most moral decisions when he has a high intuitive understanding and an ethical character. Moreover, the physician confronts a dilemma in complex situations which direct him toward an uncertainty about his duties and pave the way for his biased interventions and decisions. Under such conditions, only his internal requirements can guarantee an ethical decision making.

What are these internal requirements, and how can they be created? Psychologists believe that an individual’s personal values motivate him/her towards a pattern of behavior. All human actions have the purpose of actualizing human values and value priorities are the most essential factors for guiding attitude and behavior; therefore, behavior can be predicted if these values are known (17). Another internal factor is human affection (emotion). The last factor is strongly connected to internal requirements and is known as moral character which is considered as personality and virtuous character in psychology and philosophy, respectively.

**Internalization of ethical instructions for guiding medical students’ ethical intuitions**

One of the common concerns of all communities is to convey their values to others and future generations in the best way. One of the goals of educational and behavioral sciences is internalization which has a significant and comprehensive effect on modifying behavior. It is not necessarily a one-way process, but it can be interactive and continuous (18). During the internalization process, the individual adopts the ideals, models, beliefs, and attitudes of others (19) and accepts them as his/her own values to the extent that he/she may be unaware of their source (20). As behavior is internalized, the person feels enthusiastic towards it even when there is no extrinsic control and others are absent, and he/she also feels psychologically tense if he/she fails to perform it. 

There are various psychological explanations which justify the phenomenon of acceptance of external values over one’s own values and criteria. For instance, the theories of cognitive dissonance, self-encouragement and justification, and empathy can be pointed out in this regard (21). Moreover, an individual’s tendency to be assimilated into a group is influential on his/her acceptance of that group’s beliefs and attitudes. One of the most accepted justifications is that human beings try to keep a consistency between their behavior and attitudes, as well as between their different attitudes; hence, they convert the values that are necessary for their social life and environmental adaptation into their own values (22). The outcome of internalization is the long-term persistence of beliefs, attitudes, and behavior without an external controlling force (23). Nevertheless, the behavior that is created by an extrinsic motive such as punishment, fine, and reward will be eliminated when the external agent is removed (22).

Ethical internalization is a quality of advanced ethical growth. For example, in Kohlberg’s model of ethical development, internalization is the feature of the post-conventional stage in which an individual behaves neither for reward and punishment nor for adherence to social conventions, but acts on the basis of pure internal factors (21).

Some of the most important factors in the formation of ethical behavior that originate from the individual’s internal inclinations will be briefly discussed below. These factors include individuals’ emotions, moral and virtuous character, and attitude which can also be controllable. Moreover, the effect of law on the internalization of behavior will be discussed.

    *1.*  *The role of emotions in medical decision making*

An individual’s emotional tendencies are one of the factors that internally lead him/her to an action. Physicians’ ethical decision makings in many ethical dilemmas, particularly in relation to sensitive issues such as abortion and organ transplants, are significantly influenced by emotions (24). In previous investigations, it was observed that the effect of emotions on decisions was greater than the impact of wisdom (25). Some emotional conditions will compel physicians to think irrationally (like a child) and endanger their moral decisions (26). Therefore, in the presence of an affective factor, physicians’ selected options differ from other instance; this means that many of the precepts of theoretical ethics in the field of practice are inefficient (26). 

In the past, most moral philosophers considered emotions to be an inappropriate and also destructive basis for ethics (27). In recent centuries, philosophers have gradually assumed a greater role for emotions. For example, David Hume, in the18^th^ century, paid attention to the role of emotions in moral knowledge and took virtue into account as a sensual quality (28). He accepted the existence of a moral common sense in all people and stated that the resources for moral admiration and condemnation were sympathy and humanitarianism (28). 

Until the past few decades, psychologists had similar views and most of them considered emotion to be the reason for prejudice and bias which prevent correct judgment. In the history of psychology, most of the decision making theories emphasized the role of reason and cognition. Moreover, in the field of professional ethics, it was often believed that emotions should be omitted from the realm of professional decisions to the extent possible, particularly in professions such as medicine which deals more with people’s feelings. These theories have been greatly changed over the last century (24). 

Today, according to numerous studies that have been carried out in this area, most experts believe that although some emotions can distort understanding and cause selfish and immoral decisions, other emotions called “moral sentiments” are related to the community and welfare of others and less concerned with personal interests, and thus, important in ethical decision making (29). “Empathy” is one of the main moral sentiments and is the main factor in the relationship between the physician and the patient. Furthermore, it was shown that the presence of empathy in medicine reduces the risk of medical errors and complaint statistics by improving the physician-patient relationship (30).

There are even theories that not only stipulate the positive effects of emotions on ethical decisions, but also consider an essential and central role for them. They believe that what in particular makes a thought ethical and necessitates its being ethical is an emotional investment. Ethical experiences primarily affect emotions, and then, these moral responses lead to ethical thinking and behavior. Therefore, decreasing ethical experiences, disregarding emotions, and deficit in moral sentiments in practice can be regarded as the greatest moral hazards. Even regarding feelings that have negative moral influences and must be brought under control, we can say that the amount of their control depends on the person’s emotional saturation. Today, one of the critical issues of bioethics and one of the greatest problems of health care students’ education is the lack of emotional responses and empathy (26).

Emotions’ positive effects on ethical behavior have been demonstrated by several neuropsychological studies (31). It has been observed that patients whose feelings are partially inhibited due to treatment with chlorpromazine are more prone to unethical behavior such as deception (32). Furthermore, by studying patients with frontal cortex damage, Damasio et al. and Bechara‎ et al. showed that emotions have a major role in rational reasoning and disturbance of emotions can disrupt moral decisions (33, 34).

Based on religious teachings, moral emotions that arise from the transcendent aspect of human existence can be a tool for understanding the virtues and vices of moral and ethical decision making. The individual who possesses these emotions evaluates the values and anti-values with his/her own dignity and honor, and his/her heart can be a mirror for the manifestation of moral knowledge, so that the more innocent heart is, the more realistic moral knowledge will appear (35). Due to rapid advances in technology, the physical relationship between the physician and the patient, and consequently, their emotional connection is weakened day by day and medical structure moves towards eliminating the feelings of physicians in ethical decisions (12). 

**Controlling emotions**

Human’s emotions and feelings seem to be an involuntary and uncontrollable phenomena, but in fact they are not so. Emotions are closely related to perception, cognition, and learning, while cognition and learning are influential on emotions’ development. In fact, human emotions grow and evolve parallel to physical, psychological, and social development (36). This means that human emotions are in a raw state in the first years of life, and gradually escalate and change into perfect feelings or sublimation emotions. For example, in a perfect person like Ali Ibn Abi Talib (AS), there are no diminished feelings, but only complete sublimation emotions. In fact, the emotions and personality of a person pass through the information and characteristic channel and influence that person’s psyche which is intertwined with other parts of the spirit. Therefore, it can be said that, in choosing the reasonable ways of life, the real determining factor is sublimation emotions (37).

The way cognition influences emotions was seriously investigated for the first time by Schachter (38). He finally came to the conclusion that it is possible to change people’s emotions by influencing cognition. Schachter and Singer presented their bifactorial theory of emotion that considered the two factors of cognition and stimulus that are together responsible for arousal of emotions. Thus, we can say that we are able to control emotions by changing their cognitive components to some extent. Concurrent with Schachter’s discussions, Arnold presented a theory according to which the emotional stimuli are evaluated in different parts of the brain before they lead to emotional response. With the adoption of the evaluation structure in this theory, it can be said that our emotions are controlled by different brain areas (38).

Today, especially in the past three decades, the relationship between emotion and cognition has been widely studied and the possibility of making more ethical decisions by changing and controlling the emotions has been investigated and approved. Many of these theories were also approved by neuropsychological examinations (39, 40). 

Empathy is one of the most important moral sentiments and plays an important role in the relationship between patient and physician, and like other emotions, it has two main parts, an affective component and a cognitive component. If the level of the cognitive component is high, we can control it easier and the quality of physicians’ decisions can be improved. It also provides the opportunity for using specific trainings, creates more empathy in physicians, and improves the quality of medical decisions (29). Accordingly, today, many scholars try to enhance the feeling of empathy in physicians by focusing on the cognitive component of emotions to find ways to teach and select medical students, and to train physicians (41). For example, it was shown that empathy training caused physicians to show a 51% increase in empathy measurement criteria which was higher than the control group that had not received training in terms of empathy. According to researchers’ findings, if appropriate curricula are developed for increasing physicians’ empathy towards patients, the influence of training will be clearly observed after 6 months (30). 

Researchers have also tried to find new methods in order to achieve greater success in teaching empathy. For example, Tillman et al. presented a method that made training empathy to physicians easier and improved their learning (42).

    *2.*  *Moral and virtuous character*

Ethical and virtuous character is the other issue closely related to a person’s internal tendencies. Ethical and moral character psychology is an interpretation frequently used in ethics of psychology and the discussions of moral development. Moral character primarily focuses on the virtuous character, but they are not necessarily equivalent. In addition to virtues, other characteristics such as strong will, self-confidence, self-motivation, and a sense of control over oneself can also be important in shaping an ethical personality and being regarded as a moral character (43).

***Moral character***

In ethics of psychology, ethical development theories either place more emphasis on cognitive factors, such as Piaget’s and Kohlberg’s theories, or focus more on behavior, like social learning theory. However, in some theories like that of James Rest, not only is the ethical action mentioned, but also the necessity of forming an ethical character for repeating an ethical action and stabilizing it in adverse conditions is emphasized. Character is built by performing a particular behavior model which here includes virtuous behavior patterns. Ethical character is having the power and skill to behave in certain ways in every condition, including under pressure. Like every other personal trait, ethical character needs training and it may be different depending on each individual. Some people have stronger wills and more personal abilities, and are more self-confident. However, ethical agent must overcome opposing factors, resist pleasures, and cope with fatigue (44).

Ethical character is created though practice, and changing it into habit facilitates the occurrence of ethical behavior. Our ancestors also considered the existence of a disposition and stable character to be important in ethics, and did not think impromptu behavior to be ethical. On the basis of common definition, ethics is those stable characteristics and features in one’s ego that motivate one to act spontaneously and without thinking. In *Jame-o-Saadat*, Naraghi defines character as a sensual disposition that issues actions without the need for thinking and attitude (45). Ibnu Miskawaih uses the word “state” to mean stable features in his *Tahzib Al Aklaq wa Tahhir al A`raq* (46).

**Virtue ethics and virtuous character**

Medical ethics was previously virtue-centered. The Hippocratic Oath requires the physician to follow virtue and promotes virtues like modesty, sobriety, patience, punctuality, and piety. This oath implies that an individual’s character is as important as his behavior (47). At the present time, medical ethics is action-oriented and principle-oriented, and some doubt has recently been cast upon the effectiveness of principle-based methods. Some scholars believe that medical ethics’ solutions are abstract and general, and are not responsive to the mental and emotional aspects of health care, and thus, virtue ethics is the suitable alternative solution (48, 49).

The concept of virtue points to individuals’ character traits, and practical attitudes which include a type of motivational force for their behavior (50). In other words, virtue means people’s specific internalized practical modes leading them to immediately behave acceptably (51). In fact, virtue is a disposition which helps us to behave appropriately regarding our feelings and actions and it occurs when we are psychologically and intellectually in equilibrium (52). Virtue ethics is agent-centered and asks “how to be?” and is opposed to other normative ethics that are action-centered and ask “what to do?”. In virtue-based ethics, righteousness is more important than acting appropriately and the goodness and badness of the agent determines the actions’ correctness and wrongness (53).

In fact, virtue is a desirable personal property, its purpose is acquiring virtues and a virtuous life, and it focuses on the individual’s motivation and character traits (54). In the framework of virtue ethics, the most important factor is the individual’s motivation and character, and motivation is a crucial factor for the behavior to be ethical. However, virtue ethics is criticized for being pure virtue ethics which is Aristotle’s version of virtue ethics and is applicably weak and cannot tell us what we need to do. Furthermore, this theory is ambiguous regarding the evaluation and appropriate justification of an ethical behavior (51). In other words, the question is how a model of ethical behavior can be created for the individual by the benevolent intentions of an ethical agent. Therefore, virtue ethics needs to refer to other theories and be complemented by them. This means that virtue ethics can include concentration on duty, following ethical principles, and taking the benefit into account. Like practice-centered ethics, in this approach, which is complementary and pluralistic, the rules of ethical behavior are important, but, unlike practice-centered ethics, virtues are not instrumental and do not serve the laws (51).

In this vein, duty-based ethics and virtue ethics that are based on the doer’s character can be considered as two complementary dimensions of ethics rather than competitive and opposing dimensions. Accordingly, there is a good ethical trait which is the very willingness to act for each principle, and there is a principle which is indicative of the trait and defines the type of practice for each good ethical feature. In Frankena’s words, “principles without traits are impotent” and “traits without principles are blind” (55). 

The next question that can be posed is whether a virtuous person’s behavior pattern is duty-based or consequentialist. It seems that duty is prioritized over consequences of an action in the field of virtue ethics, while paying attention to outcome is one of the responsibilities of each person. Duty-based ethics is a more perfect stage of consequentialism regarding Kohlberg’s stages of moral development theory (35).

Another feature of virtue ethics is particularism which is highly consistent with medical ethics and avoiding the use of predefined principles. Bioethics specialists believe that solving medical problems based on case-specific theories is more harmonious with conventional moral thinking than decisions based on ethical theories, although particularism is highly criticized for relativity and it is not accepted in the field of ethics philosophy. As a result of this, the appropriateness of using general ethics rules to deal with physicians’ ethical problems has been questioned (56) and resulted in an approach named casuistry ethics which is a modified form of particularism (57) and does not completely deny ethical principles (58). There is not a consensus on the extent to which virtue ethics can be regarded as casuistry ethics (59); however, virtue ethics is more consistent with the feature that medical ethics requires to focus on details and specific cases. 

Another group of particularistic ethicists support care ethics and believe that, in the field of medical ethics, it is necessary to follow the approach which is based on care ethics since there is a strong relationship between medicine and caring (60). The theory of care ethics that was presented by Carol Gilligan for the first time is one of the new versions of virtue ethics (61). It is based on love, connection, and care for judgment and dealing with ethical issues in comparison to the dominant masculine approach in ethics which is rational and based on concepts such as justice, autonomy, and rights (61, 62).

***Teaching virtue and creating ethical character***

There are various challenges in the field of medicine regarding the issue of teaching virtue ethics and this can result in the viewpoint that the goal of teaching medical ethics cannot be the training of virtuous physicians when it is sufficient to make the physicians skilled enough to solve ethical problems. The ability to solve problems is as necessary as other medical skills, but it would be more appropriate to conduct studies to clarify how and to what extent teaching virtue is possible and how students’ ethical progress can be evaluated (63).

The most important criticism on teaching virtue is that it is not related to medicine faculties, and it is the responsibility of the church, school, and family and etc. On the other hand, some scholars believe that the virtue that is intended to be taught to medical student is related to medicine (e.g., appropriate behavior with a particular patient and how a physician must be) and cannot be conducted by other organizations. Moreover, teaching virtue is a de facto reality and medicine faculties will teach medical character and personality whether such teaching is intended to be provided or not and whether it is virtuous or vicious. Each student will select a teacher as his/her role model, and the best method for learning virtuous behavior is having a model. Therefore, it is necessary to find a way for altering the behavior of the medical model towards virtuous behavior (64).

Another criticism is related to the fact that we do not have a method for evaluating teaching virtue. How can the success of teaching be understood when the goal of teaching is having good intention, changing character, or even doing the right thing when other people are absent? Pellegrino and Thomasma believe that the students will be taught virtues or vices, regardless of whether they are measurable, during their training of basic sciences and clinical medicine. There are other skills that cannot be easily measured. Even the measurement of skills that we consider to be measurable cannot be entirely relied upon. Therefore, teaching virtue is unavoidable for this reason, and methods must be sought for evaluating and promoting it (64).

    *3.  Attitude and change of attitude*

Attitude is one of the internal factors that have been attended to in the process of change of theory into practice. In sum, attitude can be regarded as the individual’s conceptual and emotional positioning towards an issue (65). It has always been considered that accepted information is changed into attitude and eventually leads to practice. This idea has many opponents and it can only be said that attitude, to some extent, plays a part in the development of a behavior (23). In order to be changed into practice, an attitude should be associated with specific features. As an example, stronger attitudes lead to action more frequently. Each attitude consists of three cognitive, affective, and behavioral dimensions and, using various methods, can be intervened and changed by these dimensions (66).

Several studies with various approaches and methods have indicated that physicians’ attitudes can be changed and their behavior can be improved through different trainings in practical situations (67-69). For instance, critical changes in the education of residents and use of student-centered methods of teaching has changed their attitude towards patients infected with AIDS which, in turn, has led to the improvement of medical services provided for them (70). In summary, by directing instructions towards creating influential changes in a person’s attitude, more behavioral modifications can be witnessed.

Another factor that is closely related to attitude is faith. Faith is accompanied by a sort of fondness, holiness, and transcendence of values and is followed by the desire to act. Faith has extraordinary practical influences and its investigation in relation to religious groups is beneficial and even essential (71, 72).

    *4.*  *Appropriate application of law and administrative solutions*

Law is an external factor that forces the individual into ethical behavior, and it can internalize the behavior in some conditions. Law is an important supportive factor of behavior and can have an important role in actualization of ethics. It is even said that collective wisdom and sense of human responsibility cannot be trusted with administering justice and some degree of law enforcement will be required, especially for the relationships between two groups (73). Moreover, law provides a suitable environment for ethical behavior and increases the cost of unethical behavior, in addition to decreasing the cost of ethical behavior. Its other important effect is helping people to abandon their unethical habits and form other habits (50). One of the reasons of unethical behavior is an individual’s inability to control him/herself. Disciplinary regulations that foster individuals’ self-control and self-regulation lead to the internalization of ethics through actualization (18).

However, the intervention of law in the field of ethics needs more attention and delicacy. First, the exercise of power and use of legal sanctions without providing the necessary cultural backgrounds lead to the formation of extrinsic behavior. Participation of the stakeholders in regulation of laws is the necessary requirement for individuals to obey them. It is necessary that the individual understands why laws exist and is convinced that the existence of these laws is essential for group life and beneficial to all (74).

Second, although strict laws control behavior very well, they make the behavior extrinsic. The clarification that psychology suggests is that human beings always try to justify and clarify their own behavior. If there is an excessive external force to change the behavior, the individual will relate his/her behavior change to that force and when the external power does not exist, he/she will not follow that behavior anymore. However, if these laws are not too strict, the individual will connect the behavior to his/her own desire, and therefore, when there is no external monitor, he/she will feel required to behave that way again (18).

Therefore, it is essential to clarify and justify individuals through appropriate training of laws and creating necessary cultural substructures. In this regard, they can even participate in making laws or be counseled, and the laws should not be strict to the extent possible. On the other hand, intrinsic motivation is converted into extrinsic motivation, and as a result, the behavior turns into its former state when the law is omitted or unable to act.

During the process of teaching physicians, these points must be taken into consideration while making regulations and suggesting solutions. Furthermore, the medical education system is an appropriate platform for creating the cultural infrastructure of medical regulations and its achievement is greatly influential in the internalization of the regulations of medical society. 

## Conclusion

Due to its importance, medicine needs members who follow its professional principles. These requirements are taught to the physicians during medical training courses, but most of them are not put into practice. To bridge this gap, the sense of following these essential principles can be created by both regulative monitoring and internalization within physicians. Due to the fact that law cannot replace physicians’ internalized codes, internalizing these instructions must be considered as a part of medical education. Internalization is a complex issue to discuss and has been debated in psychology, sociology, and philosophy. The current study has tried to explain the factors effective on ethical internalization and suggest solutions for narrowing the gap between ethical theory and practice by pointing to the issue and stating its necessity. These solutions are mainly medical education-centered and are as follows:

1. Controlling emotions by education
2. Teaching virtue and creating ethical character
3. Changing attitude by education
4. Modifying legal and administrative solutions

Medical education authorities’ attention and acceptance of this issue and its application in curriculum design procedures will be a great step towards putting medical ethics into practice. However, given the structure of modern medicine, this task requires extensive efforts. Therefore, more investigations are required in order to generalize the internalization model in medical education.
